# Consensus-Based Recommendations for Assessing Post-Intensive Care Syndrome: A Systematic Review

**DOI:** 10.3390/jcm14103595

**Published:** 2025-05-21

**Authors:** Helmar Bornemann-Cimenti, Johanna Lang, Sascha Hammer, Kordula Lang-Illievich, Sebastian Labenbacher, Stefan Neuwersch-Sommeregger, Christoph Klivinyi

**Affiliations:** 1 Department of Anaesthesiology and Intensive Care Medicine, Medical University of Graz, 8010 Graz, Austria; helmar.bornemann@medunigraz.at (H.B.-C.); sascha.hammer@medunigraz.at (S.H.); sebastian.labenbacher@medunigraz.at (S.L.); 2 Department of Anesthesiology, Perioperative Medicine and General Intensive Care Medicine, Paracelsus Medical University Salzburg, 5020 Salzburg, Austria; 3 Department of Anaesthesia and Intensive Care Medicine, State Hospital Güssing, 7540 Güssing, Austria; 4 Department of Anaesthesiology and Intensive Care Medicine, General Public Hospital of the Brothers of St. John of God, 9300 St. Veit/Glan, Austria

**Keywords:** post-intensive care syndrome, consensus, practice guidelines, patient outcome assessment, critical care outcomes

## Abstract

**Background:** Post-intensive care syndrome encompasses physical, cognitive, and psychological impairments that persist in patients after discharge from an intensive care unit. There is considerable variation in the tools used for assessment. This systematic review aimed to summarize the consensus-based recommendations for assessing post-intensive care syndrome. **Methods:** A comprehensive literature search identified four consensus-based guidelines. A quality assessment carried out using the Appraisal of Guidelines for Research and Evaluation II tool demonstrated high methodological standards across all the included papers. **Results:** The guidelines consistently emphasize assessing cognition, mental health, and physical function as the core domains. However, there are notable differences in the specific tools recommended. Major et al. focused on physical examinations, while Mikkelsen et al. proposed a fundamental package of five tools covering the key domains. Spies et al. aimed for a pragmatic set of freely available instruments administrable within 30 min. Nakanishi et al. provided a detailed ranking of instruments for each domain. The availability of validated translations varied considerably across languages. Some tools developed specifically for post-intensive care syndrome were not considered by any consensus conference. **Conclusions:** Further work is needed to establish a universally accepted standard for assessing post-intensive care syndrome that considers practical implementation across diverse settings and languages.

## 1. Introduction

Post-intensive care syndrome (PICS) is a clinically defined condition that encompasses a spectrum of physical, cognitive, and psychological impairments that persist in patients after their discharge from an intensive care unit (ICU). The term was formally established during a stakeholder conference convened by the Society of Critical Care Medicine to raise awareness of the long-term consequences of critical illness and ICU treatment [1]. A recent meta-analysis of more than 10,000 cases revealed a pooled incidence of 54%, with an increased rate in patients who had longer ICU stays [2]. The risk factors were grouped as person-related (e.g., previous mental or cognitive problems), disease-related (e.g., organ dysfunction), and ICU-related parameters (e.g., sedative use, respiratory support) [3].

PICS is characterized by new or worsening impairments in three primary domains: physical, cognitive, and mental health [4,5]. Large-scale studies have revealed alarming statistics. A study from the Netherlands with more than 2300 ICU survivors (including those who underwent urgent and elective surgery and medical ICU patients) found that fatigue affects 30% of ICU survivors; depression and anxiety impact 13% and 12%, respectively; and 5 to 7% experience frailty and post-traumatic stress disorder (PTSD) [6]. In surgical patients, these numbers tend to be even higher. A database analysis involving nearly 800,000 ICU survivors revealed comparable results for anxiety and depression [7].

The burden of PICS extends beyond mental health issues, with physical symptoms playing a significant role in patients’ recovery. In particular, pain emerges as a persistent challenge, affecting between one-third and one-half of patients even a year after hospital discharge [8,9]. This prolonged experience of pain, combined with mental health challenges and potential cognitive impairments, creates a multifaceted struggle for ICU survivors. The prevalence and persistence of these symptoms highlight the need for comprehensive, long-term care strategies to address the diverse needs of PICS patients and support their recovery process.

As even small temporal disturbances in biopsychosocial factors were shown to have a detrimental effect on wellbeing [10,11], it is obvious that long-term changes can lead to significantly reduced quality of life and increased healthcare utilization among survivors [12,13]. Quality of life has a significant correlation with perceived disability [14]. In the long term, PICS frequently results in delayed return to work or worsened employment status [15]. One-third of ICU survivors are unemployed one year following hospital discharge [16]. This figure demonstrates the immense socio-economic relevance of this topic. Furthermore, the impact of PICS extends beyond the individual patient and also affects family members, a phenomenon referred to as post-intensive care syndrome-family (PICS-F) [17]. Family members often experience their own psychological distress, including anxiety and depression, as they cope with the aftermath of a loved one’s critical illness [18].

The literature on PICS has sharply increased within the past five years [19]. However, recent reviews have demonstrated large heterogeneity in the scales and measures used to assess symptoms and severity [20,21]. Therefore, defining core outcome sets is a crucial step to increase comparability for different aspects, e.g., clinical efficacy, quality management, and, of course, scientific evaluation.

Consensus conferences play a pivotal role in shaping clinical practice by systematically evaluating the available evidence and synthesizing expert opinions [22,23]. The process involves several key steps, including task definition, participant identification and recruitment, information gathering, consensus building, and result dissemination. These conferences bring together diverse perspectives from various disciplines and specialties, ensuring a comprehensive examination of the topic at hand. By employing structured group decision-making techniques, consensus conferences foster productive discussions and mitigate the potential dominance of individual viewpoints, ultimately leading to more balanced and well-informed recommendations [23]. The value of consensus conferences becomes particularly evident in complex medical fields that demand interdisciplinary expertise. In such areas, the collective expertise of a diverse group is far more reliable and comprehensive than individual judgments. By bridging the gap between evidence-based medicine and expert opinion, consensus conferences contribute to the advancement of medical knowledge and the improvement of patient care.

In the field of PICS, there are several consensus-based approaches to define diagnostic tools and instruments. The aim of this study is to summarize these consensus-based recommendations for using diagnostic tools to assess PICS, with special emphasis on the methodological quality, the recommended tools, and the availability or restriction of access by licenses or languages.

## 2. Methods

We conducted a systematic review of the literature for consensus-based recommendations. The terms used to search the PubMed, Ovid Medline, Embase, and PsycNet databases are outlined below (Table 1).

The inclusion criterion was the reporting of consensus-based recommendations of tools for assessing PICS. Studies using methodologies other than the consensus-based recommendation (e.g., clinical trials) were excluded.

These primary matches were manually assessed for duplicates. Screening was performed by two independent researchers. If the assessments conflicted, the publications were carried forward to the next round. A third reviewer was involved in the final step.

The references of relevant review articles were screened for additional sources. Furthermore, a search was carried out on Google Scholar to identify further sources.

The data were extracted into a Microsoft Excel spreadsheet. Different recommendations were narratively compared. Quality was assessed by two independent reviewers using the AGREE II tool developed by the International Appraisal of Guidelines, Research and Evaluation (AGREE) research team [24]. This tool is regarded as the gold standard for evaluating the quality of guidelines and is endorsed by several healthcare organizations [25,26].

All the recommended tools were reviewed for the availability of validated translations to the most frequently spoken languages. According to the CIA’s World Fact Book, these are English (18.8% of the world population), Mandarin Chinese (13.8%), Hindi (7.5%), Spanish (6.9%), and French (3.4%) [27].

Our protocol was pre-registered in the open science framework (https://osf.io/u4sbp, accessed on 12 May 2025). This study was prepared by following the PRISMA guidelines [28]. No new data were created or analyzed in this study.

## 3. Results

**Figure 1:**
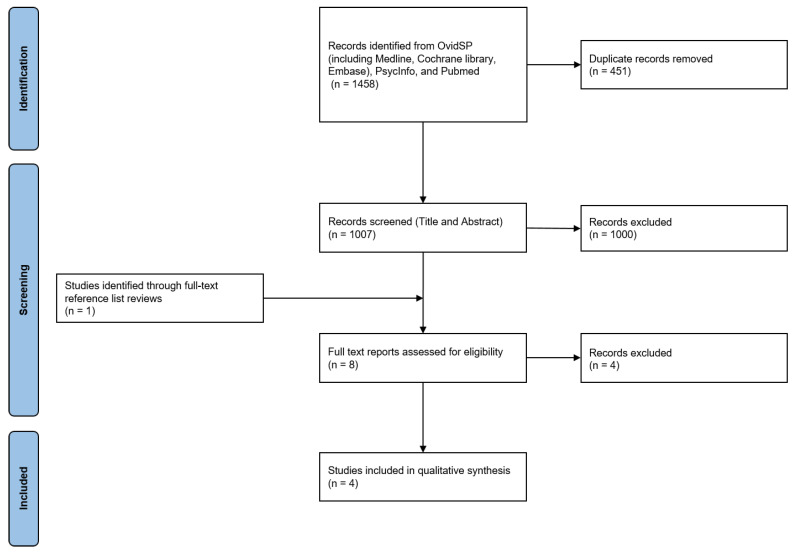
A flow chart of the literature screening process.

The last search of the literature was conducted in March 2025. The initial screening resulted in 1458 primary hits. After removing duplicates and screening abstracts and full texts, three papers were included. The screening of the gray literature revealed one additional article, resulting in four articles being included in this review. Figure 1 presents a flow chart of this study.

Two articles followed the Delphi protocol, while two others reflected the proceedings of consensus meetings. Three or four consensus rounds were carried out. The number of participants varied between 10 and 31. Anesthetists or intensive care physicians, nurses, and physiotherapists were included in all the committees. Further details are presented in Table 2, and the included instruments are listed in Table 3.

The quality assessment was carried out by two independent researchers, and all the guidelines received an overall recommendation. The detailed values are presented in Table 4.

Only a minority of the recommended tools were publicly available: 5 out of 22 were freely available without any restriction, and 15 were free with minor restrictions (available upon request), at least for academic or non-commercial purposes. However, two of the tools must be purchased for access.

There were considerable differences between the availability of validated translations in different languages. A total of 91% of the tools were available in Chinese, while only 63% were translated into Hindi (Table 5).

## 4. Discussion

The lack of a universally established standard for assessing post-intensive care syndrome (PICS) remains a significant challenge in critical care medicine despite its importance being recognized over a decade ago [33]. While experts agree on the necessity of a set of screening instruments for mental health (e.g., anxiety, depression, and PTSD), cognitive impairment (e.g., executive function, memory, attention, visuospatial skills, and mental processing speed), and physical impairment (e.g., pulmonary, neuromuscular, and physical function), the implementation of a standardized assessment protocol has proven elusive. This gap in standardization hinders the ability to compare research findings across different studies and healthcare settings, potentially impeding progress in understanding and treating PICS. As research in this field continues to evolve, there is an urgent need for collaborative efforts among healthcare professionals, researchers, and policymakers to establish a standardized, comprehensive, and widely applicable assessment protocol for PICS, which would significantly enhance patient care and advance the scientific understanding of this syndrome.

Our systematic literature review identified four consensus-based recommendations regarding tools that should be utilized in assessing PICS [17,18,19,20]. All the included publications followed a high methodological standard, which resulted in high-quality assessment scores. However, it is important to note the consistently low scores obtained across all the tools in the “applicability” section.

All the consensus groups, consisting of a multidisciplinary board of experts, systematically reviewed the available literature. While nurses, physiotherapists, and intensive care physicians or anesthesiologists were represented in all the studies, patients’ representatives were only included in a single consensus group [30]. These multidisciplinary boards were carefully assembled to ensure a comprehensive approach to the review process, drawing on diverse expertise and perspectives. The systematic literature review allowed for a thorough examination of the existing research and evidence in the field. However, the limited inclusion of patients’ representatives in most consensus groups raises questions about the extent to which patient perspectives and experiences were considered in the decision-making process. This underrepresentation of patient voices may impact the overall applicability and patient-centeredness of the resulting recommendations or guidelines.

The recommendations are derived from a variety of tools that were previously established and validated for specific sub-aspects associated with PICS. The EuroQol-5, utilized for evaluating health-related quality of life, emerges as the sole instrument endorsed by all four studies. The grip strength and six-minute walk tests were recommended in three out of four guidelines. This consensus indicates that these tools may serve as foundational elements in the development of standardized assessment protocols for PICS. All the other tools received recommendations from a maximum of two groups. Although all the recommended tools previously demonstrated effectiveness in various diseases, none have been specifically validated for PICS patients. Given that this population differs from others in certain biopsychosocial components and exhibits significant heterogeneity, it is worth considering the extent to which experiences from other populations can be validly transferred.

Historically, the initial recommendation was formulated by Major et al. [29]. They convened a group of international scientific experts who participated in a Delphi process. While the primary objective of this working group was to establish recommendations for physiotherapy for survivors of intensive care, they also formulated a set of measurement tools as a core outcome measure set (COMS). These recommendations emphasize physical examinations, including dynamometry and spirometry. This approach is distinctive, as no other guideline incorporates these specific measures. Appleton et al. estimated the incidence of muscular weakness to be 40% in ICU patients [34]. From this perspective, the inclusion of a muscular weakness assessment is highly justifiable. However, recent evidence from a broad range of populations suggests a correlation between grip strength and spirometry parameters [35]. Consequently, the inclusion of both tests may not be necessary.

Mikkelsen et al. were the first in this field to establish a recommendation that was endorsed by an international, educational, and scientific society [30]. The Society of Critical Care Medicine organized a consensus conference in 2019 to address the prediction and identification of long-term impairments following critical illness. The conference convened 31 participants representing a diverse array of professional backgrounds and academic disciplines, fostering an interdisciplinary exchange of knowledge, perspectives, and expertise. The participants proposed assessment tools for four key domains: long-term cognition, mental health, physical function, and PTSD. These assessments are intended to be administered 2–4 weeks post-hospital discharge to patients deemed to be at risk for PICS based on a functional evaluation conducted at the time of discharge. Their defined outcome measures, covering five key domains, can be considered a core framework for this purpose. Although there may be variations in the specific tools recommended by different authors, the core domains addressed in this set are consistently recognized across recommendations in the field.

Spies et al. aimed to establish a pragmatic and comprehensive set of assessment tools for clinical evaluation [31]. Their primary objective was to ensure that the selected instruments were both freely available and capable of being efficiently administered within a 20–30 min timeframe, thereby optimizing clinical utility. In cases necessitating more thorough and nuanced analysis, they proposed a secondary set of more extensive tools to complement the initial assessment. Their recommendations’ notable deviations from other established protocols are primarily attributable to the self-imposed constraint of utilizing only freely accessible instruments, which significantly influenced their selection criteria. Notably, Spies et al. were the sole research group to exclude the widely used Hospital Anxiety and Depression Scale from their assessment battery, a decision necessitated by the scale’s licensing restrictions [36]. This exclusion highlights the challenges researchers face when balancing the need for comprehensive assessment with practical considerations, such as accessibility and cost-effectiveness.

Furthermore, Spies et al. distinguished themselves as the only research team to assess the practicality and feasibility of their proposed measurement set through direct application with actual patients. This empirical approach to validating their assessment protocol demonstrates a commendable commitment to ensuring the real-world applicability and effectiveness of their recommended tools in clinical settings. Such practical validation enhances the credibility and potential adoption of their assessment framework in diverse healthcare environments.

The latest recommendations were formulated by members of the Japanese Society of Intensive Care PICS Committee [32]. Nakanishi et al. followed a slightly different approach, as they not only focused on recommending specific tools but also generated a detailed ranking. They reviewed the literature for instruments used in clinical trials and rated them according to their perceived usefulness. They listed the top three instruments for every domain, i.e., physical, cognitive, mental, ADL, and quality of life (QoL). Furthermore, they specifically assessed sleep and pain, as well as the mental health and QoL of family members [32]. Notably, they were the sole group to incorporate at least one aspect of PICS-F, which is remarkable given the critical role of family involvement in alleviating spiritual distress [37]. Furthermore, there is no universally accepted standard for recording data in this sub-area. A recent review by Hayes et al. highlights the absence of a unified outcome set for PICS-F, underscoring the ongoing need for further development in this domain [38].

Likewise, the spiritual component of PICS has not been incorporated into any existing assessment tools. Drawing on insights from fields such as palliative care, it is evident that this aspect warrants increased attention [39,40,41]. Recent studies indicate that many patients with PICS experience unmet spiritual and social support needs within their social environments [40]. This underscores the necessity of considering PICS within a more comprehensive bio-psycho-social-spiritual framework. Future research should prioritize this holistic approach to more accurately reflect patients’ lived experiences. Furthermore, definitions and assessment instruments should be revised to encompass these critical dimensions.

We assessed the availability of validated translations in the five most frequently spoken languages for all the mentioned tools. Some of them are only available in English, limiting their recommendation in an international context. For example, more than one-third of the recommended tools are not available in Hindi, the third most frequently spoken language in the world, with more than 600 million speakers [27]. Therefore, it is crucial that these tools are translated and validated in additional languages.

The licensing restrictions associated with many tools and scales in research present significant challenges for their widespread adoption and utilization. While few instruments are freely accessible without limitations, the majority require users to submit formal requests or restrict free usage to academic or non-commercial endeavors. These constraints can lead to reduced implementation across various sectors, hindering the potential for comprehensive comparability and broader acceptance of research findings.

The approach taken by Spies et al., focusing on the use of publicly available instruments, serves as a commendable model for addressing these challenges [31]. By prioritizing open-access tools, researchers can foster greater transparency, reproducibility, and collaboration within the scientific community. This strategy not only democratizes access to research instruments but also promotes the development of standardized methodologies that can be easily adopted and replicated across different studies and disciplines. As the research landscape continues to evolve, embracing such open approaches may become increasingly crucial for advancing knowledge and ensuring equitable participation in scientific endeavors.

It is worth mentioning that some tools were specifically developed for diagnosing PICS [42,43]. Although they were tested in large cohorts and were both internally and externally validated, they were not considered by any of the consensus conferences.

Our work not only highlights the differences between individual assessment tools but also emphasizes that defining a core set of measures influences the therapy approach, as recorded deficits should be specifically optimized. Therefore, considerations such as the inclusion of muscle strength have far-reaching consequences, not just affecting diagnosis.

### Limitations

Our literature search also revealed consensus-based guidelines that were not considered in this systematic review, as they did not specifically focus on PICS or were only formulated for a specific subpopulation. Needham et al. defined core outcome measures for patients after respiratory failure [44]; Hodgson et al. focused on patients after extracorporeal membrane oxygenation [45]; and Haywood concentrated on core outcomes after cardiac arrest [46]. Most of the domains in these recommendations are close or comparable to those in the studies we selected for our systematic review. However, as our research focused on evaluating consensus statements specifically on PICS, we decided not to include them.

## 5. Conclusions

Consensus-based recommendations are crucial for defining core outcome sets to assess PICS. Our systematic review identified four consensus-based recommendations. All of them consistently emphasize assessing cognition, mental health, and physical function. However, notable differences exist between the individual recommendations. Therefore, which recommendations should be followed depends on the specific scope and circumstances (e.g., academic vs. clinical settings, the availability of translations, and licensing). Future work should also take greater account of the bio-psycho-social-spiritual dimension of this disease.

## Figures and Tables

**Table 1:** The search strategy for the PubMed and Ovid databases.

PubMed	(“post intensive care syndrome”[All Fields] OR “postintensive care syndrome”[All Fields] OR “PICS”[All Fields] OR “post ICU syndrome”[All Fields]) AND (“guideline”[Publication Type] OR “guidelines as topic”[MeSH Terms] OR “guideline”[All Fields] OR “consens*”[All Fields] OR “consensus”[MeSH Terms] OR “consensus”[All Fields] OR “statement”[All Fields] OR “statements”[All Fields] OR “delphi”[All Fields] OR “societies”[All Fields] OR “society”[All Fields] OR “meeting”[All Fields] OR “conference”[All Fields] OR “experts”[All Fields])
Ovid	((“post intensive care syndrome” or “postintensive care syndrome” or “PICS” or “post ICU syndrome”) and (“guideline” or “consens*” or “consensus” or “statement” or “statements” or “delphi” or “societies” or “society” or “meeting” or “conference” or “experts”)).mp. [mp=ti, ot, ab, tx, kw, ct, sh, fx, hw, tn, dm, mf, dv, kf, dq, bt, nm, ox, px, rx, an, ui, sy, ux, mx]

**Table 2:** Methodological details of included studies.

Author	Major, 2016 [29]	Mikkelsen, 2020 [30]	Spies, 2021 [31]	Nakanishi, 2023 [32]
Methodology	Delphi consensus process	Consensus development conference	Semi-structured consensus meeting	Delphi consensus process
Type of attendance	Online	Online and in person	In person	Online
Systematic review of the literature	Yes	Yes	Yes	Yes
Number of rounds	3	4	3	3
Number of participants	10	31	14	23
Nationalities	International	International	Germany	Japan
Intensive care physician/anesthesiologist	Yes	Yes	Yes	Yes
Nurse	Yes	Yes	Yes	Yes
Physio-, ergo-, occupational therapist	Yes	Yes	Yes	Yes
Psychologist, psychiatrist	Yes	Yes	Yes	No
Patients’ representative	No	Yes	No	No
Other specialties	No	Internal medicine, general practitioner, pharmacist, rehabilitation expert, advanced practice provider	Sepsis researchers, telehealth researcher, respiratory specialist, healthcare manager	No

**Table 3:** Instruments included in recommendations.

	Major, 2016 [29]	Mikkelsen, 2020 [30]	Spies, 2021 [31] (Initial Assessment)	Spies, 2021 [31] (Extended Assessment)	Nakanishi, 2023 [32]
Cognitive dysfunction	MMSE, Subjective Global Assessment Tool	MoCA	Mini-cog, animal naming	RBANS, TMT	MoCA, MMSE, SMQ
Anxiety and depression	HADS	HADS	PHQ-4	PHQ-8, GAD-7	HADS
Post-traumatic stress disorder	IES-R	IES-R, IES-6	No recommendation	IES-R	IES-R
Physical function	6MWT, 4 m time walk/gait speed, cycle ergometry testing, DEMMI, TUG, MRC	6MWT	TUG	2MWT, SPPB	6MWT, MRC-score,
Physical examination	Handgrip strength and handheld dynamometry, maximum inspiratory pressure, maximum expiratory pressure	No recommendation	Handgrip strength	Handgrip strength	Handgrip strength
Activities of daily living	FIM, SPPB, SF-36 (physical function domain), Barthel Index, Katz ADL, Lawton IADL	No recommendation	No recommendation	No recommendation	Barthel Index, IADL, FIM
Quality of life	SF-36, EQ-5D	EuroQol-5D-5L	EQ-5D-5L	WHODAS 2.0, EQ-5D-5L	SF-36, EQ-5D-5L, EQ-5D-3L, EQ-VAS, SF-12
New or worsening health problems	No recommendation	No recommendation	No recommendation	Single items and NRS of subjective mental and physical health	No recommendation
Fatigue	MFI	No recommendation	No recommendation	No recommendation	No recommendation
Nutrition	SNAQ, MUST	No recommendation	No recommendation	No recommendation	No recommendation
Sleep	RCSQ	No recommendation	No recommendation	No recommendation	PSQI
Pain	VAS for pain	No recommendation	No recommendation	No recommendation	BPI

Barthel Index: Barthel Index of Activities of Daily Living; BPI: Brief Pain Inventory; DEMMI: De Morton Mobility Index; EQ-5D: EuroQol 5-Dimension Questionnaire; EQ-5D-3L: EuroQol 5-Dimension 3-Level Questionnaire; EQ-VAS: EQ-5D Visual Analog Scale; FIM: Functional Independence Measure; GAD-7: Generalized Anxiety Disorder Scale-7; HADS: Hospital Anxiety and Depression Scale; IADL: Lawton Instrumental Activities of Daily Living Scale; IES-R: Impact of Event Scale—Revised; IES-6: Impact of Event Scale—6-item short form; Katz ADL: Katz Index of Independence in Activities of Daily Living; Mini-Cog: Mini-Cognitive Assessment Instrument; MFI: Multidimensional Fatigue Inventory; MMSE: Mini-Mental State Examination; MoCA: Montreal Cognitive Assessment; MRC: Medical Research Council score; MUST: Malnutrition Universal Screening Tool; NRS: numeric rating; PHQ-4: Patient Health Questionnaire-4; PHQ-8: Patient Health Questionnaire-8; PSQI Scale: Pittsburgh Sleep Quality Index; QoL: quality of life; RBANS: Repeatable Battery for the Assessment of Neuropsychological Status; RCSQ: Richard Campbell Sleep Questionnaire; SF-12: Short Form-12 Health Survey; SF-36: Short Form-36 Health Survey; SMQ: Short-Memory Questionnaire; SNAQ: Short Nutritional Assessment Questionnaire; SPPB: Short Physical Performance Battery; TMT: Trail Making Test; TUG: Timed Up and Go Test; VAS: visual analog scale; WHODAS 2.0: World Health Organization Disability Assessment Schedule 2.0; 2MWT: 2-Minute Walk Test; 6MWT: 6-Minute Walk Test.

**Table 4:** A quality assessment according to the Agree-II Tool. The values present the percentage of the maximum possible score within each domain.

	Major, 2016 [29]	Mikkelsen, 2020 [30]	Spies, 2021 [31]	Nakanishi, 2023 [32]
Scope and purpose	88.9	100.0	100.0	94.4
Stakeholder involvement	66.7	72.2	83.3	77.8
Rigor of development	50.0	62.5	64.6	60.4
Clarity of presentation	50.0	55.6	50.0	50.0
Applicability	22.2	22.2	33.3	22.2
Editorial independence	100.0	100.0	100.0	100.0
Overall quality	66.7	83.3	66.7	66.7

**Table 5:** Licenses and availability of translations of the tools recommended in the guidelines.

Domain	Tool	License	English	Mandarin Chinese	Hindi	Spanish	French
Cognition	Montreal Cognitive Assessment (MOCA)	Free upon request, requires training session	Yes	Yes	Yes	Yes	Yes
	Mini-Mental State Examination (MMSE)	Commercial	Yes	Yes	Yes	Yes	Yes
	Short memory questionnaire (SMQ)	No data	Yes	No	No	No	No
	MiniCog	Free upon request	Yes	Yes	Yes	Yes	Yes
	Animal naming	Free	Yes	Yes	No	No	No
	Subjective Global Assessment Tool	Free	Yes	Yes	No	Yes	Yes
Anxiety and Depression	Hospital Anxiety and Depression Scale (HADS)	Free upon request	Yes	Yes	Yes	Yes	Yes
	Impact of Event Scale—Revised (IES-R)	Free upon request	Yes	Yes	Yes	Yes	Yes
	Patient Health Questionnaire-9 (PHQ-9)	Free	Yes	Yes	Yes	Yes	Yes
	Patient Health Questionnaire-4 (PHQ-4)	Free	Yes	Yes	No	Yes	Yes
PTSD	Impact of Event Scale—Revised (IES-R)	Free upon request	Yes	Yes	Yes	Yes	Yes
Physical function	Medical Research Council score (MRC)	Free with credit line	Yes	No	No	No	No
QoL	Short Form-36 Health Survey (SF-36)	Free with credit line	Yes	Yes	Yes	Yes	Yes
	EuroQol 5-Dimension Questionnaire (EQ-5D-5L)	Free upon request	Yes	Yes	Yes	Yes	Yes
	EuroQol 5-Dimension Questionnaire (EQ-5D-3L)	Free upon request	Yes	Yes	Yes	Yes	Yes
	EuroQuol-VAS	Free upon request	Yes	Yes	Yes	Yes	Yes
	Short Form-12 Health Survey (SF-12)	Commercial	Yes	Yes	Yes	Yes	Yes
	World Health Organization Disability Assessment Schedule 2.0 (WHODAS 2.0)	Free for non-commercial use	Yes	Yes	No	Yes	No
Function	Barthel Index	Free for non-funded academic users	Yes	Yes	Yes	Yes	Yes
	Instrumental Activities of Daily Living (IADL)	Free upon request	Yes	Yes	No	Yes	Yes
	Functional Independence Measure (FIM)	Free	Yes	Yes	No	Yes	Yes

## Data Availability

No new data were created or analyzed in this study.
